# Hypermethylation of DMTN promotes the metastasis of colorectal cancer cells by regulating the actin cytoskeleton through Rac1 signaling activation

**DOI:** 10.1186/s13046-018-0958-1

**Published:** 2018-12-04

**Authors:** Ya-Ping Ye, Hong-Li Jiao, Shu-Yang Wang, Zhi-Yuan Xiao, Dan Zhang, Jun-Feng Qiu, Ling-Jie Zhang, Ya-Li Zhao, Ting-Ting Li, Li-Liang, Wen-Ting Liao, Yan-Qing Ding

**Affiliations:** 1 Department of Pathology, Nanfang Hospital, Southern Medical University, Guangzhou, 510515 China; 2 Department of Pathology, School of Basic Medical Sciences, Southern Medical University, Guangzhou, 510515 China; 3 Guangdong Provincial Key Laboratory of Molecular Tumor Pathology, Guangzhou, China

**Keywords:** Colorectal cancer, DMTN, Metastasis, RAC1 signaling, Hypermethylation

## Abstract

**Background:**

Colorectal cancer (CRC) is one of the most common digestive malignant tumors, and DMTN is a transcriptionally differentially expressed gene that was identified using CRC mRNA sequencing data from The Cancer Genome Atlas (TCGA). Our preliminary work suggested that the expression of DMTN was downregulated in CRC, and the Rac1 signaling pathway was significantly enriched in CRC tissues with low DMTN expression. However, the specific functions and underlying molecular mechanisms of DMTN in the progression of CRC and the upstream factors regulating the downregulation of the gene remain unclear.

**Methods:**

DMTN expression was analyzed in CRC tissues, and the relationship between DMTN expression and the clinicopathological parameters was analyzed. In vitro and in vivo experimental models were used to detect the effects of DMTN dysregulation on invasion and metastasis of CRC cells. GSEA assay was performed to explore the mechanism of DMTN in invasion and metastasis of CRC. Westernblot, Co-IP and GST-Pull-Down assay were used to detect the interaction between DMTN and ARHGEF2, as well as the activation of the RAC1 signaling. Bisulfite genomic sequence (BSP) assay was used to test the degree of methylation of DMTN gene promoter in CRC tissues.

**Results:**

We found that the expression of DMTN was significantly decreased in CRC tissues, and the downregulation of DMTN was associated with advanced progression and poor survival and was regarded as an independent predictive factor of CRC patient prognosis. The overexpression of DMTN inhibited, while the knockdown of DMTN promoted, invasion and metastasis in CRC cells. Moreover, hypermethylation and the deletion of DMTN relieved binding to the ARHGEF2 protein, activated the Rac1 signaling pathway, regulated actin cytoskeletal rearrangements, and promoted the invasion and metastasis of CRC cells.

**Conclusion:**

Our study demonstrated that the downregulation of DMTN promoted the metastasis of colorectal cancer cells by regulating the actin cytoskeleton through RAC1 signaling activation, potentially providing a new therapeutic target to enable cancer precision medicine for CRC patients.

**Electronic supplementary material:**

The online version of this article (10.1186/s13046-018-0958-1) contains supplementary material, which is available to authorized users.

## Introduction

Colorectal cancer (CRC) is one of the most common digestive malignant tumors, and over the past decade, the CRC incidence rate has declined because of the gradual increase in colonoscopy examinations. In contrast to the rapid decline in overall CRC incidence, the rates in individuals aged younger than 55 years have increased by almost 2% per year from the mid-1990s to 2014, with changes in lifestyle and diet [1, 2]. It is well known that metastasis is the leading cause of death in patients with malignant tumors, and metastatic tumors are found in 40–50% of patients at initial diagnosis and treatment [3–6]. The metastasis of CRC is a complex process and is regulated by both oncogenes and suppressor genes. However, at present, more research is focused on the function of metastasis-related oncogenes, while relatively few studies have addressed the mechanism of metastasis-related suppressor genes [7, 8]. Therefore, it is essential to elucidate the function and molecular mechanisms of more metastasis-related suppressor genes underlying the metastasis of CRC from multiple regulatory levels using multiomics analysis.

An increasing number of studies have shown that multiple genetic and epigenetic changes are required for carcinogenesis and the progression of CRC [9–11]. We now have an initial view of the landscape of genetic alterations, including microsatellite instability (MSI), chromosomal instability (CIN) and methylations, which occur in CRC [12]. However, the biological functions of these features and how they initiate and promote the tumorigenesis and progression of CRC is largely unknown. Therefore, it is imperative to explore the characterization of transcriptomic subtypes of CRC and to identify genes with required expression for the proliferation and metastasis of CRC cells [13].

DMTN is a transcriptional differentially expressed gene (DEG) that was identified using CRC mRNA sequencing data from The Cancer Genome Atlas (TCGA), and it maps to a region of the short arm of human chromosome 8p21.1, encoding the DMTN protein [14]. DMTN is an actin-binding/bundling protein that was originally isolated from the human erythrocyte membrane, which directly binds F-actin through actin binding sites to regulate cytoskeleton remodeling [15, 16]. The actin cytoskeletal rearrangements occur upon activation of the small family of Rho GTPases, RhoA, Rac1 and Cdc42, and the activity of the GTPases is regulated by guanine nucleotide exchange factors (GEFs), GTPase activating proteins (GAPs) and guanine dissociation inhibitors (GDIs) [17–19]. Rho-Rac guanine nucleotide exchange factor 2 (ARHGEF2), which activates Ras homolog family member A (RHOA) and RAC1, has been implicated in various cellular processes involving the establishment of cell polarity, including epithelial tight junction formation and endothelial permeability [18], and it is required for oncogenic RAS signaling [20]. It is known that dysregulation in the structure and function of the cytoskeleton is an important factor in the development and progression of malignant tumors [21–24], and the abnormal expression of actin-related proteins is also similar to the malignant phenotype of the transformation of normal cells [25, 26].

DMTN is responsible for maintaining the shape and integrity of erythrocyte [25, 27–29]. Biochemical characterization of DMTN shows that it exhibits phosphorylation-dependent actin bundling activity [30]. DMTN is expressed predominantly in hematopoietic (erythrocytes, platelets, and lymphocytes), cardiac, vascular, endothelial, epithelial, skeletal and muscle components, as well as in kidney cells [14, 15]. The broad expression of DMTN suggests that it may play an important role in the regulation of the actin cytoskeleton in nonerythroid cells [31].

Some studies have shown that the dysregulation and deletion of DMTN are closely related to the carcinogenesis and metastasis of cancer. The DMTN gene maps to chromosome 8p21.1, a region that is often accompanied by the loss of heterozygosity in prostate cancer patients [32]. In prostate cancer PC-3 cells, the overexpression of DMTN restores epithelioid cell morphological phenotypes. Recent studies have shown that DMTN regulates cell shape, motility, and wound healing by modulating RhoA activation [15]. Our preliminary work suggested that DMTN expression was downregulated in CRC, and the result of a Gene Set Enrichment Analysis (GSEA) assay also suggested that the Rac1 signaling pathway was significantly enriched in CRC tissues with low DMTN expression. Considering the above results, we speculate that the dysregulation of DMTN may function as a tumor suppressor gene by regulating the activity of Rac1 signaling in the carcinogenesis, invasion and metastasis of CRC, which may be similar to the effect of truncated mutant adenomatous polyposis coli (APC) on the activation on Rac1 by relieving binding with Asef and Asef2 [33, 34]. However, the specific functions and molecular mechanisms of DMTN in the progression of CRC and the mechanisms responsible for the downregulation of DMTN remain unclear.

Therefore, in this study, we detected DMTN expression in CRC tissues, analyzed the relationship between DMTN expression and the clinical pathologic parameters, illustrated the role and associated molecular mechanism of DMTN in the invasion and metastasis of CRC, and explored the upstream regulatory mechanisms underlying the downregulation of the DMTN gene.

## Materials and methods

### Clinical specimen

The clinical research on the samples was performed according to written approval obtained from the Southern Medical University Institutional Board (Guangzhou, China). All the specimens were collected with the informed consent of the patients. Paraffin-embedded archived CRC tissue samples (*n* = 200) were collected between 2008 and 2012, and 50 fresh CRC tissues and matched adjacent normal tissues were obtained between 2014 and 2015 from the Department of Pathology, Southern Medical University. The surgically resected tissues were frozen in liquid nitrogen immediately until future analysis. The medical records of the patients were reviewed for acquisition of the clinicopathological information, including age, gender, differentiation, and TNM stage. The survival data were available for the cohort of 200 patients, and the median follow-up time was 65 (range, 3–87) months.

### Cell culture, vector construction and lentivirus infection

The human CRC cell lines were purchased from The Global Bioresource Center (ATCC, USA). HCT15 was cultured in RPMI 1640 medium (Gibco, Grand Island, NY, USA) containing 10% fetal bovine serum (Gibco, Grand Island, NY, USA). The SW620 and SW480 cells were cultured in Leibovitz’s L-15 medium supplemented with 10% FBS (Gibco). The HT29 and HCT116 cells were cultured in McCoy’s 5a medium modified with 10% FBS (Gibco). The Ls174t cells were cultured in Dulbecco’s modified Eagle’s medium (DMEM; Gibco) with 10% FBS (Gibco). All cells were cultured at 37 °C in 5% CO2. The vector construction and lentivirus infection were conducted according to previously described methods [35, 36]. Further details are provided in the Additional file 1: Supplementary Materials and Methods section.

### Transwell, wound-healing assay and three-dimensional morphogenesis assay

The transwell, wound-healing assay and the three-dimensional morphogenesis assay were conducted according to previously described methods [35, 36]. Further details are provided in Additional file 1.

### Real-time quantitative PCR, western blotting and immunohistochemistry

Real-time quantitative PCR (RT-PCR), western blotting (WB) and immunohistochemistry (IHC) were conducted according to previously described methods [35, 36]. Further details are provided in Additional file 1.

### Immunofluorescence

The cells were fixed, permeabilized, and blocked using 4% paraformaldehyde for 10 min, 0.5% Nonidet-P40 (Bioshop) for 20 min, and 10% FBS/PBS for 1 h. The antibody incubations were performed in blocking solution for 1 h, and the slides were mounted in Immuno-mount medium (Thermo Fisher Scientific). The immunofluorescence images were collected using the Olympia Deconvolution fluorescence microscope and softWoRx software (Applied Precision). The images were collected using a 100 × or 60 × 1.4 NA oil objective (Olympus).

### Co-immunoprecipitation (co-IP) assay

A total of 1 × 10^6^ cells were seeded on a 10-cm plate, and the DMTN plasmid was transfected into the CRC cells. After a 48-h incubation, the cells were washed with cold PBS. Then, ice-cold RIPA buffer was added to the plate, and the cells were scraped with a precooling spatula. The suspension was transferred to a new EP tube, shaken for 15 min and then centrifuged for 15 min at 1400 g at 4 °C. Protein An agarose was added to the protein (100 μl protein A agarose/1 ml protein). After 10 min of shaking at 4 °C, the samples were centrifuged for 15 min at 1400 g at 4 °C to remove the protein G beads. Then, anti-flag antibodies were incubated overnight with rotation at 4 °C. Protein A was added the following day to capture the antigen-antibody complexes. The complexes were incubated overnight with rotation at 4 °C or were rotated for 1 h at room temperature. After washing with wash buffer, the samples were centrifuged 6 times at 1400 g at 4 °C for 2 min each. The samples were boiled for 5 min and then washed with SDS-PAGE loading buffer. Finally, the samples were separated by SDS-PAGE and analyzed by immunoblotting.

### GST-fusion proteins and GST-pull-Down assay

The pGEX-4 T-2 plasmid (Amersham) was used to construct the vectors expressing the GST-DMTN-GEF-binding and GST-DMTN-ARHGEF2-DH fusion proteins. The pcDNA3.1-DMTN and pcDNA3.1-ARHGEF2 plasmids served as the templates. The PCR conditions were 95 °C/1 min, 18 cycles of 95 °C/30 s, 55 °C/1 min, and 68 °C/1 min. After digestion, the fragments were subcloned individually in frame with respect to GST into pGEX-4 T-2. GST-DMTN-GEF-binding and GST-DMTN-ARHGEF2-DH were expressed and purified according to the manufacturer’s instructions (Pierce Biotechnology). The purified proteins were used as bait protein for the pull-down assay.

### GTPase activation assay

GST-PBD (p21-binding domain of PAK) was used for the Rac1 activity assays [17]. The CRC cells were transfected with DMTN overexpression, knockdown or control vector. After 72 h, the cells were washed twice with ice-cold PBS and lysed in ice-cold Mg^2+^ lysis buffer. The cell lysates were centrifuged for 5 min at 13,000 g at 4 °C, and 40 μl of the supernatant was removed to determine the total Rac1 levels. The remaining supernatants were incubated with GST-PBD on glutathione-sepharose beads and rotated at 4 °C for 2 h. The beads were washed extensively in lysis buffer, and the bound proteins were separated by SDS-PAGE and immunoblotted with anti-Rac1 antibodies.

### Bisulfite genomic sequence (BSP) assay

The DMTN CpG island was searched in the NCBI Gene database and the UCSC Genome Browser on the Human February 2009 Assembly (hg19; ref. [18]). The genomic DNA was treated with sodium bisulfite and subjected to PCR, using primer sets designed to amplify the regions of interest (Additional file 1: Table S3). Bisulfite-sequencing analysis was carried out.

### Orthotopic mouse metastatic model

A surgical orthotopic implantation mouse model of CRC was performed as previously described [37]. The cells (2 × 10^6^ per mouse) were subcutaneously injected into the right dorsal flank of female BALB/c athymic nude mice (4–6 weeks of age, 18–20 g) obtained from the Animal Center of Southern Medical University, Guangzhou, China. Two weeks later, the animals were sacrificed, and the tumors were excised. A portion of the tumor was fixed in 10% formaldehyde, embedded in paraffin, cut into 5 μm sections and subjected to IHC using an anti-DMTN antibody (Abcam, Cambridge, MA, USA) or hematoxylin-eosin (H&E) staining. Another portion of the tumor was divided into small pieces of approximately 1 mm in diameter. The surgical orthotopic implantation of the CRC tumor fragments was performed in nude mice after anesthesia. The mice were killed 100 days after surgery, individual organs were excised, and metastases were observed by histological analysis. The orthotopic mouse metastatic model was carried out according to previously described methods [35, 36]. All mice were housed and maintained under specific pathogen-free conditions and used in accordance with institutional guidelines with approval by the Use Committee for Animal Care. Further details are provided in Additional file 1.

### Statistical analysis

The data were analyzed using SPSS 19.0 for Windows. The Mann-Whitney U-test and Spearman’s correlation analyses were applied to analyze the relationship between the expression of DMTN and the clinicopathological features of the CRC cases. A two-tailed paired Student’s t-test was used to compare the two experimental groups. The 5-year overall survival curves were plotted by the Kaplan-Meier method and compared with the log-rank test. A Cox proportional hazard regression model was established for the multivariate analysis of the combinatorial contribution of DMTN and the clinicopathological features to the survival of the patients. *P* < 0.05 was considered significant.

### Accession numbers for the data sets

The GEO database (GSE17538, GSE17536, GSE17537, and GSE16125) and the TCGA data were used to analyze the relationship between the expression of DMTN and the 5-year overall survival of the CRC patients. The GEO databases (GSE13294, GSE32896, GSE13067, GSE7208, and GSE35896) were used for the GSEA analysis of the “KEGG_COLORECTAL CANCER”, “RHO_GTPASES” and “Rac1 signaling pathways” gene sets in the study.

## Results

### The downregulation of DMTN correlates with advanced progression and a poorer prognosis in CRC

**Fig. 1:**
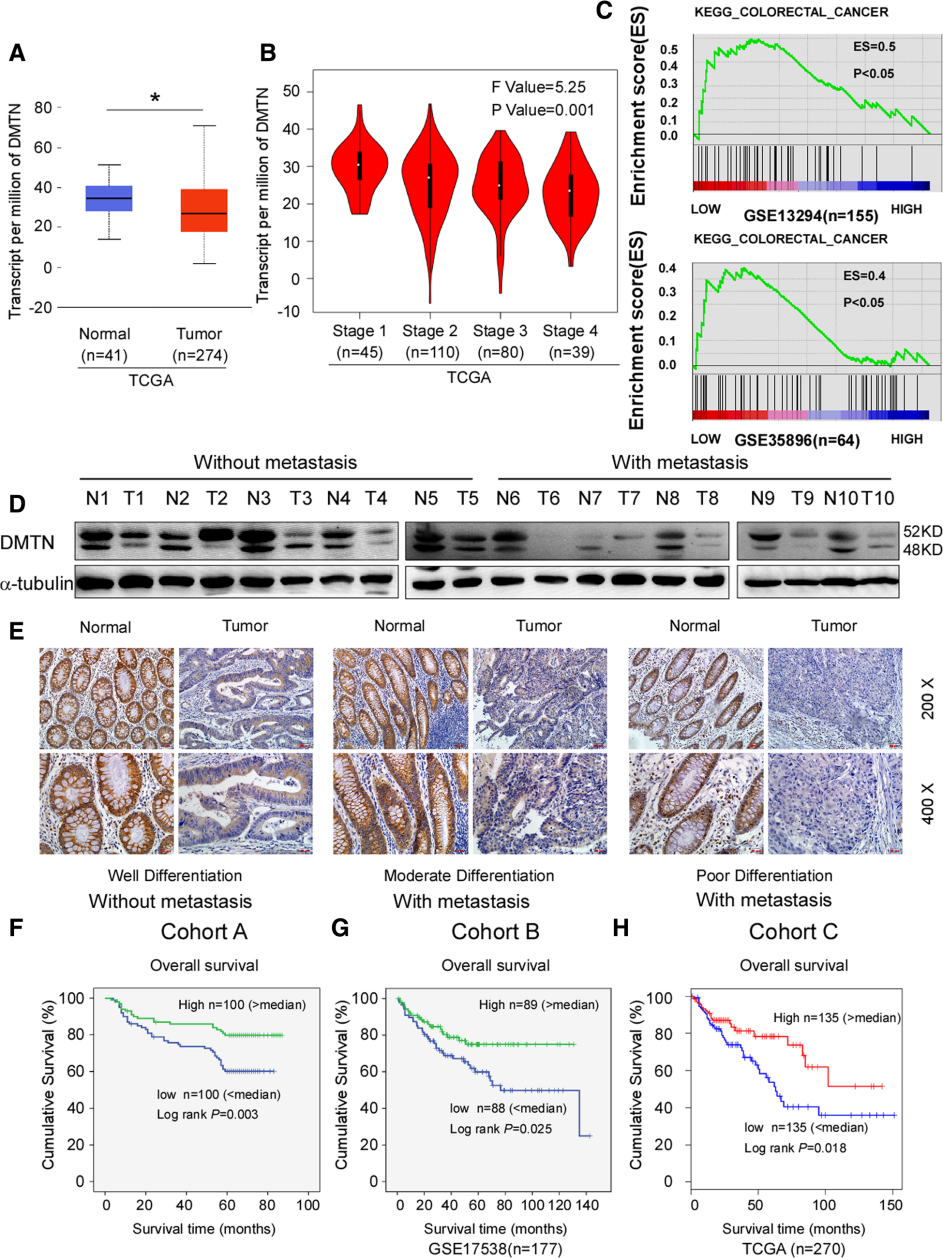
Downregulation of DMTN correlates with advanced progression and poorer prognosis in CRC. **a**, The expression of DMTN in CRC tissues and in normal colon tissues in the TCGA mRNA sequencing data. **b**, The relationship between DMTN expression and TNM stage in the TCGA data. **c**, GSEA analysis of the “KEGG_COLORECTAL CANCER” gene set in the low versus high expression group of DMTN in CRC. D, Western blot analysis of DMTN expression in 10 fresh human CRC tissues with different degrees of differentiation (N = normal, T = tumor). **e**, IHC analysis of DMTN expression in 200 paraffin-embedded archived human CRC tissues (representative results). **f**, **g**, **h**, Kaplan-Meier analyses of CRC patient outcomes with low versus high expression of DMTN in our data (Cohort A), GSE17538 (Cohort B) and GSE17538 (Cohort C) (*P* < 0.05, log-rank test). * *P* < 0.05

The TCGA database was first used to analyze the expression of DMTN in CRC tissues. The results showed that the expression of DMTN in CRC tissues was significantly downregulated compared with that in normal colon tissues (Fig. 1a, *P* < 0.05), and the expression level of DMTN gradually decreased with the increase in stage (Fig. 1b, *P* < 0.05). The analysis from Oncomine (https://www.oncomine.org/) and The Human Protein Atlas (https://www.proteinatlas.org/) also showed the same results (Additional file 1: Figure S1A and 1B). Moreover, the results from the GSEA analysis suggested that the CRC-related gene set, “KEGG_COLORECTAL CANCER”, was significantly enriched in CRC tissues with low DMTN expression (Fig. 1c, ES = 0.4, *P* < 0.05). These results indicate that DMTN may function as a tumor suppressor in the progression of CRC.

Next, RT-PCR, western blot and IHC were used to detect the expression levels of DMTN in 50 fresh and 200 paraffin CRC samples. The western blot and RT-PCR results suggested that the mRNA and protein levels of DMTN were lower in CRC tissue than in the paired normal colon tissue, especially in cases with metastasis (Fig. 1d; Additional file 1: Figure S1C, *P* < 0.05). The IHC results showed positive DMTN expression mainly localized in membranes and cytoplasm, as marked by yellow-brown staining, and the expression of DMTN in CRC tissue was downregulated by 79.5% (169/200) in CRC patients (Fig. 1e). Furthermore, the relationship between DMTN expression and clinicopathological parameters was analyzed. The results showed a negative correlation between DMTN and the various differentiations, Dukes stage, and TNM stage (Additional file 1: Table S4, *P* < 0.05). Kaplan-Meier survival analysis revealed a significantly longer 5-year overall survival rate in patients with CRC with high DMTN expression than in patients with low DMTN expression, and the 5-year overall survival rate declined with decreasing DMTN expression (Fig. 1 f, g, h and Additional file 1: Figure S1D; log-rank, *P* < 0.05). DMTN was regarded as an independent predictive factor for the prognosis of patients with CRC (Additional file 1: Table S5).

### Exogenous DMTN knockdown promotes the invasion and metastasis of CRC cells, and the upregulation of DMTN inhibits the invasion and metastasis of CRC cells

**Fig. 2:**
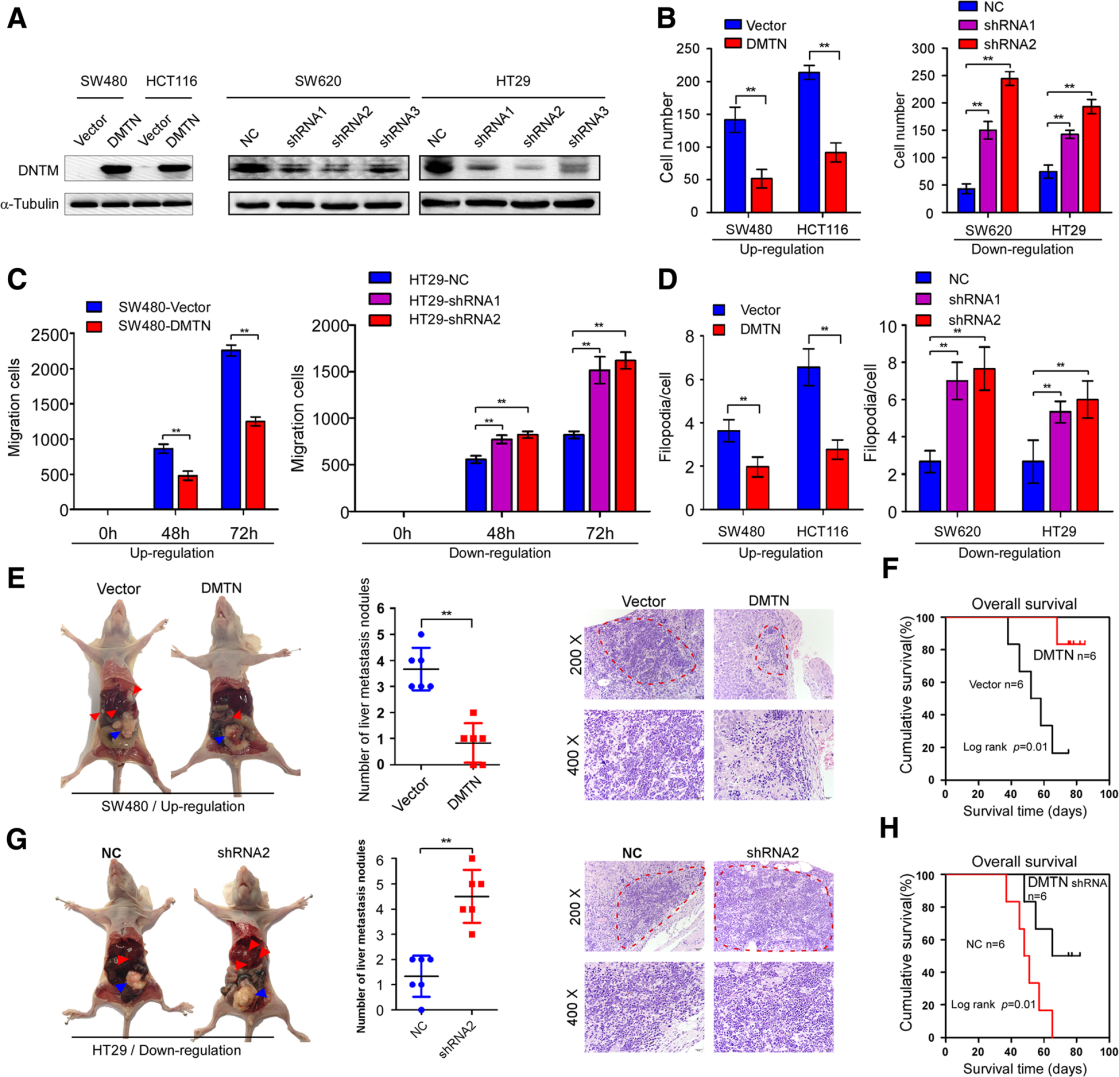
Exogenous DMTN knockdown promotes the metastasis of CRC cells, and the upregulation of DMTN inhibits the metastasis of CRC cells. **a**, Western blot analysis of the overexpression and knockdown of DMTN in CRC cell lines. **b**, **c**, **d**, Transwell invasion assay, scratch wound healing and 3-D cell culture analysis of the migration of CRC cells with DMTN overexpression or knockdown. **e**, **g**, Representative gross images of the intestines and livers from the DMTN overexpression and knockdown groups in the cecum orthotropic transplantation assay (left). The arrows show metastases in the intestines (blue) and livers (red). The scatter plot shows the number of liver metastatic nodules observed in each group (middle). The liver sections were stained with H&E (right). **f**, **h**, Overall survival time of the DMTN overexpression and knockdown groups (log-rank test, *P* < 0.05). The error bars represent the mean ± SD of 3 independent experiments, ** *P* < 0.01

Next, we detected the effects of DMTN overexpression or knockdown on the invasion and metastasis of CRC cells. Lentiviral vectors were used to establish stable expression cell lines, and the western blot results showed that stable expression CRC cell lines were successfully established (Fig. 2a and Additional file 1: Figure S2A). The transwell migration assay results showed that the migration cell numbers of CRC cells with DMTN overexpression decreased markedly compared with those of the control group, while the migration cell numbers of CRC cells with DMTN knockdown increased dramatically (Fig. 2b and Additional file 1: Figure S2B, *P* < 0.05). The scratch wound-healing assay results demonstrated a slower speed of wound healing in CRC cells with DMTN overexpression than in the control group, while the speed of wound healing was faster in CRC cells with DMTN knockdown than in the control group (Fig. 2c and Additional file 1: Figure S2C, *P* < 0.05). The 3-D cell culture assay results revealed a marked decrease in the number of protrusions in CRC cells with DMTN overexpression compared with that in the control group, while the number of protrusions in CRC cells with DMTN knockdown dramatically increased (Fig. 2d and Additional file 1: Figure S2D, *P* < 0.05).

Moreover, exogenous DMTN overexpression or knockdown was performed to test the effects on the metastasis of CRC cells in vivo. The results from the orthotopic mouse metastatic model showed that the overexpression of DMTN decreased tumor numbers and the frequency of liver metastasis (Fig. 2e, *P* < 0.05), and DMTN overexpression also extended the overall survival time of the nude mice injected with the CRC cell lines (Fig. 2f, *P* < 0.05). Conversely, the knockdown of DMTN by shRNAs enhanced the metastatic potential of CRC cells (Fig. 2g, *P* < 0.05) and shortened the overall survival of the mice (Fig. 2h, *P* < 0.05).

### The downregulation of DMTN enhances the activity of the Rac1 signaling pathway by relieving binding with the ARHGEF2 protein

**Fig. 3:**
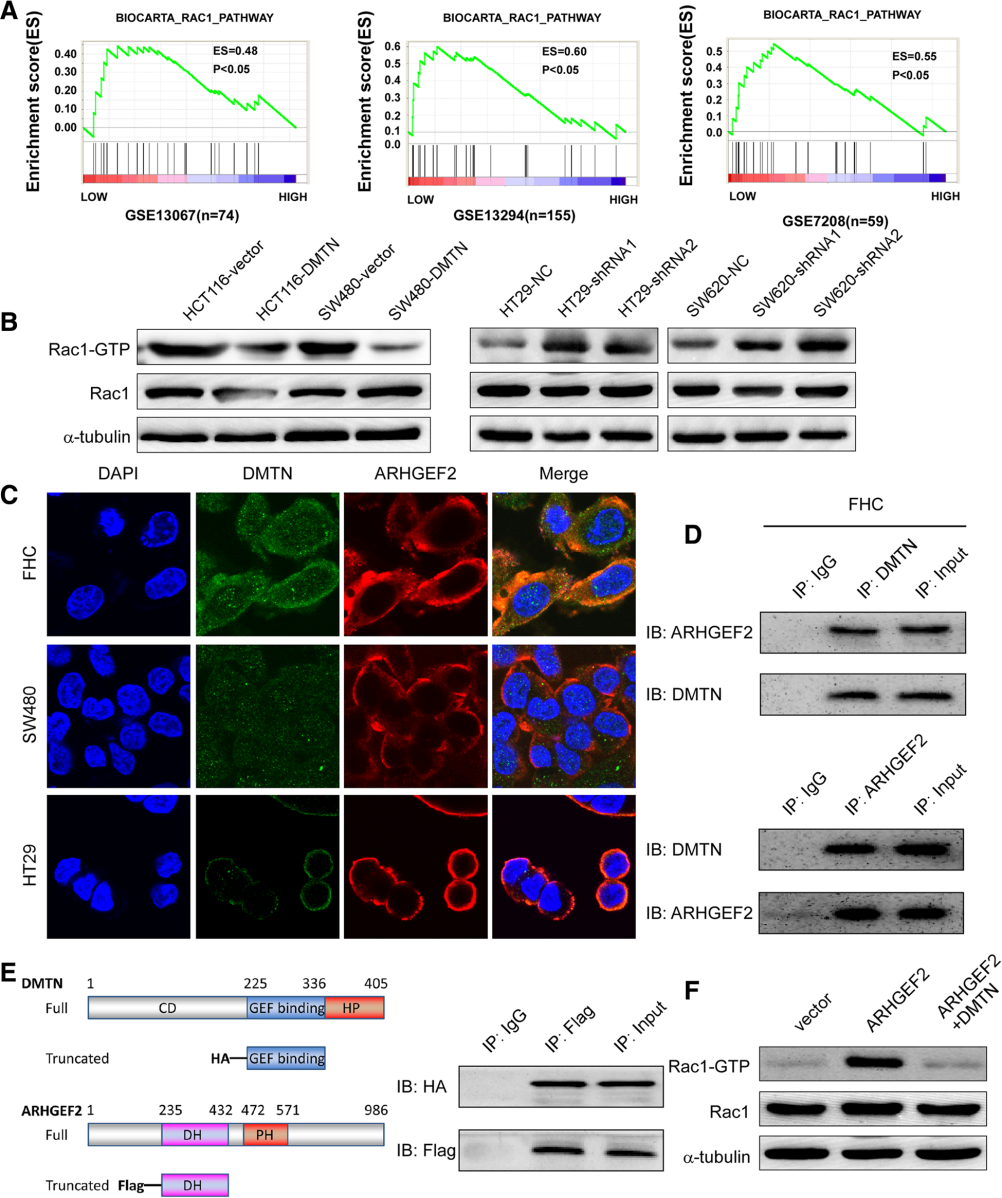
Downregulation of DMTN enhances the activity of the Rac1 signaling pathway by relieving binding to the ARHGEF2 protein. **a**, GSEA analysis of the “Rac1 signaling pathways” gene set in the low versus high expression group of DMTN in CRC using the GEO data. **b**, Western blot analysis of the expression of Rac1-GTP in the CRC cell lines with DMTN overexpression or knockdown. **c**, Immunofluorescence detection of the colocalization between DMTN with ARHGEF2 in CRC-immortalized cells (FHC) and CRC cells (SW480 and HT29). **d**, Coimmunoprecipitation (Co-IP) analysis of the protein interaction between DMTN with ARHGEF2 in CRC-immortalized cells (FHC). **e**, GST-pull-down detection of the interaction between the DMTN core domain (with an HA tag) and the ARHGEF2 DH domain (with a flag tag). **f**, Western blot analysis of the activity of Rac1 in the SW480 cells cotransfected with the ARHGEF2 and DMTN vectors

To explore the molecular mechanisms of DMTN in the invasion and metastasis of CRC, we first analyzed the signaling pathways that might be regulated by DMTN through the GSEA assay. The results suggested that the “RHO_GTPASES” and “Rac1 signaling pathways” gene sets were significantly enriched in CRC tissues with low DMTN expression in GSE13067, GSE13294 and GSE7208 (Fig. 3a and Additional file 1: Figure S3A, *P* < 0.05). The western blot results also showed decreased the expression of Rac1-GTP in HCT116 and SW480 cells with DMTN overexpression compared with that in the control group, while the expression of Rac1-GTP was increased in HT29 and SW620 cells with DMTN knockdown compared with that in the control group (Fig. 3b, *P* < 0.05).

Although the precise mechanism of Rho GTPase (RhoA, Rac1 and Cdc42) suppression by DMTN is unclear, several plausible and hypothetical models can be proposed. DMTN may bind to and inhibit GEF activity, form an inactive complex with GDI-RhoA-GDP, or enhance GAP function. We used protein interaction prediction software based on protein structure (PPI, https://bhapp.c2b2.columbia.edu/PrePPI/) and mass spectrometry (MALDI-TOF-MS) to analyze and detect DMTN-interacting proteins (Additional file 1: Table S6 and S7). The results showed that DMTN interacted with a member of the GEF family, ARHGEF2, which was significantly upregulated in the CRC tissue (Additional file 1: Figure S3B). The results of immunofluorescence showed that DMTN colocalized with ARHGEF2 in the cell membrane and cytoplasm in the CRC-immortalized cells FHC. The results of the Co-IP and GST-pull-down assays further indicated that the DMTN core domain (226-336aa) interacted with the ARHGEF2 DH domain (Fig. 3d, e). Moreover, the western blot results further revealed the increased expression of Rac1-GTP in SW480 cells transfected with the ARHGEF2 vector compared with that in the control vector, while the expression of Rac1-GTP was decreased in SW480 cells cotransfected with the ARHGEF2 and DMTN vectors (Fig. 3f).

### The downregulation of DMTN promotes actin cytoskeletal rearrangements in CRC cells through the Rac1 signaling pathway

**Fig. 4:**
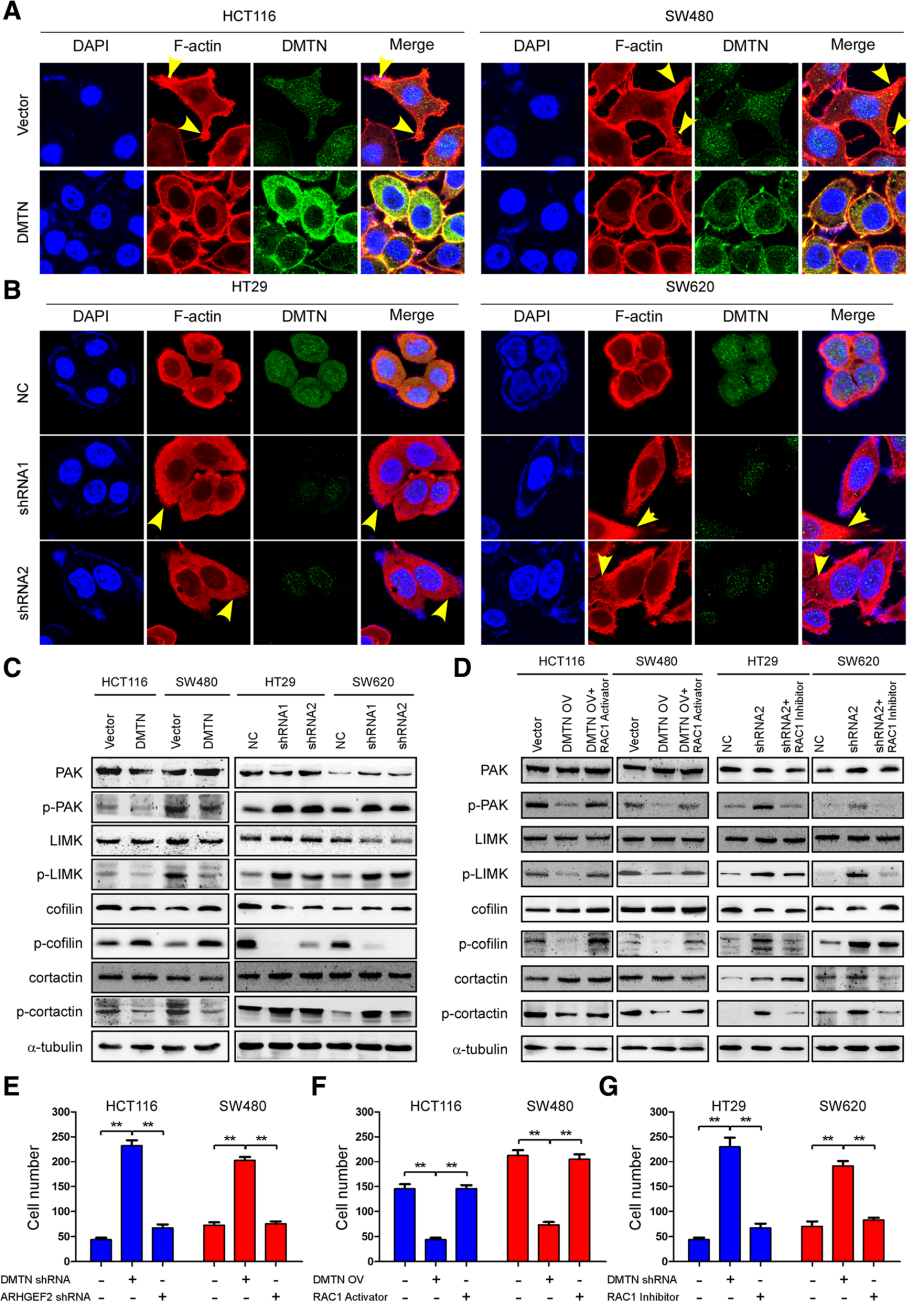
Downregulation of DMTN promotes actin cytoskeletal rearrangements in CRC cells through the Rac1 signaling pathway. **a**, **b**, Confocal laser scanning microscopy analysis of the actin cytoskeletal remodeling of CRC cells with DMTN overexpression or knockdown (arrows show cell lamellipodia and protrusion formation). **c**, Western blot detection of the expression of the target genes of Rac1 signaling in the CRC cells with DMTN overexpression or knockdown. **d**, The effect of DMTN-ARHGEF2-Rac1 on the expression of target genes using a Rac1 signaling inhibitor and activator by western blot analysis. **e**, Rescue experiment analysis of the effect of DMTN-ARHGEF2-Rac1 on the migration of CRC cells with DMTN overexpression transfected with the ARHGEF2 shRNA vector. **f**, **g**, Rescue experiment analysis of the effect of DMTN-ARHGEF2-Rac1 on the migration of CRC cells with DMTN overexpression using a Rac1 signaling activator or inhibitor. The error bars represent the mean ± SD of 3 independent experiments, ** *P* < 0.01

Cell motility and actin cytoskeletal rearrangements occur upon the activation of the small family of Rho GTPases, RhoA, Rac1 and Cdc42. We investigated the effect on actin cytoskeletal remodeling of Rac1 activation induced by DMTN downregulation. The results from the confocal laser scanning microscopy assay indicated that the HCT116 and SW480 cells with DMTN overexpression showed a round morphology and fewer lamellipodia and protrusions compared with the control group, which showed an elongated morphology and more protrusions, while HT29 and SW620 cells with DMTN knockdown showed an elongated morphology and more lamellipodia and protrusions compared with the control group, which showed a round morphology and fewer protrusions (Fig. 4a and b). We next detected the expression of target genes of Rac1 signaling. Western blot analyses indicated that enforced DMTN suppressed the phosphorylation of PAK, LIMK, and Cortactin but increased that of Cofilin in HCT116 and SW480 CRCs (Fig. 4c).

We further tested the effect of DMTN-ARHGEF2-Rac1 on the expression of target genes using a Rac1 signaling inhibitor and activator. The western blot results showed that decreasing the expression of p-PAK, p-LIMK, and p-Cortactin and increasing the expression of p-Cofilin in HCT116 and SW480 cells with DMTN overexpression was reversed by treatment with the Rac1 signaling activator, while the increasing expression of p-PAK, p-LIMK, and p-Cortactin and the decreasing expression of p-Cofilin in the HT29 and SW620 cells with DMTN knockdown was reversed by treatment with the Rac1 signaling inhibitor (Fig. 4d). The result of the rescue experiment also demonstrated a reversal of the increasing number of migrating HT29 and SW620 cells with DMTN knockdown by transfection with the ARHGEF2 shRNA vector (Fig. 4e). Moreover, the decreasing number of migrating HCT116 and SW480 cells with DMTN overexpression was reversed by treatment with Rac1 signaling activator (Fig. 4f), while the increasing migrating number of HT29 and SW620 cells with DMTN knockdown was reversed by treatment with Rac1 signaling inhibitor (Fig. 4g).

### Epigenetic regulation of the DMTN gene through changes in the methylation status of the gene promoter

**Fig. 5:**
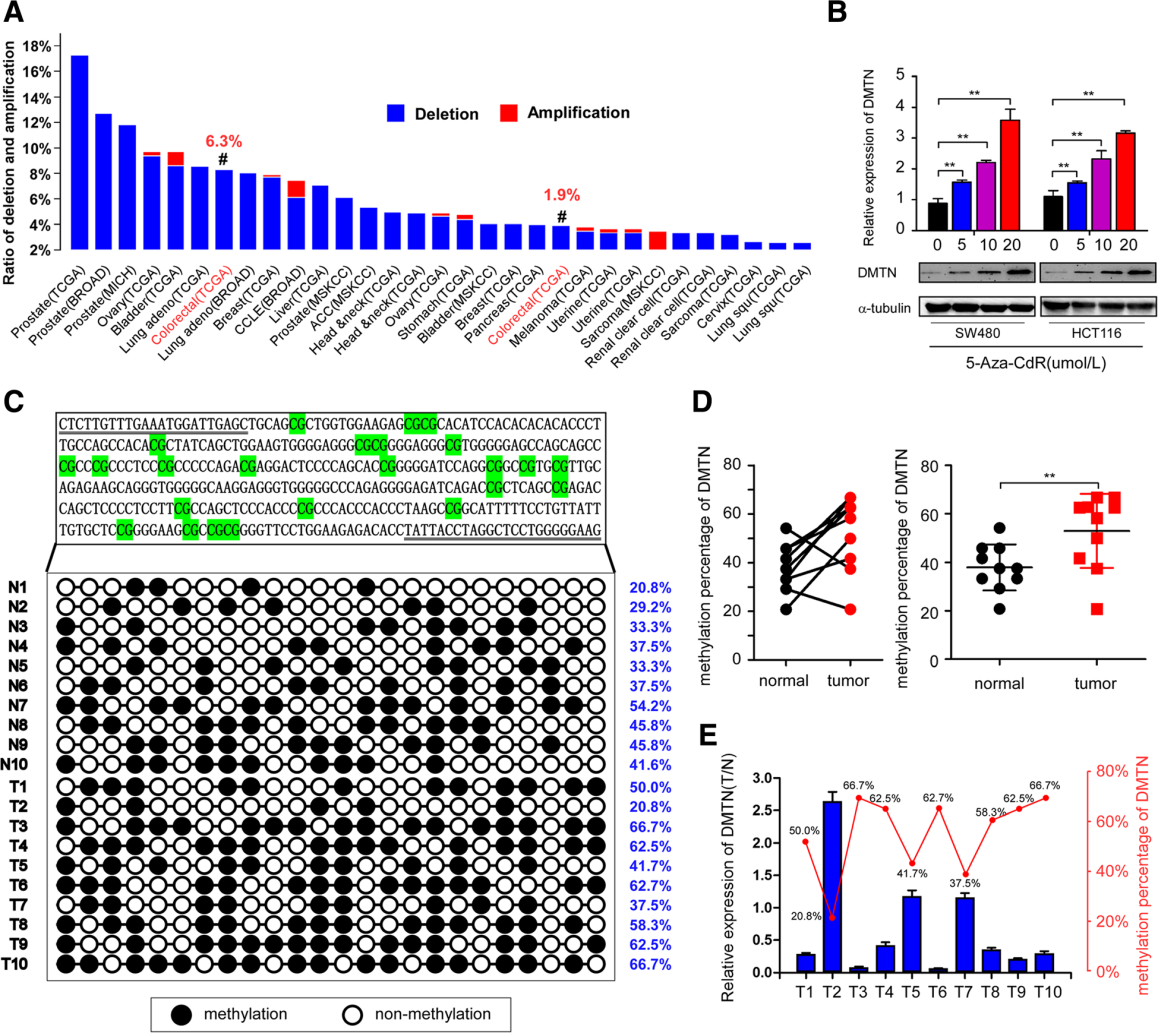
Epigenetic regulation of the DMTN gene through changes in the methylation status of the gene promoter. **a**, Analysis of the deletion rate of the DMTN gene in CRC using the TCGA exon sequencing data. **b**, The expression of DMTN in CRC cells treated with 5-Aza-CdR by RT-PCR and western blot analysis. **c**, **d**, Detection of the degree of DMTN promoter methylation in 10 CRC tissues and the paired normal tissues through the bisulfite genomic sequence (BSP) assay (N = normal, T = tumor). **e**, The relationship between the expression of DMTN and the degree of DMTN promoter methylation based on the results of both RT-PCR and bisulfite genomic sequence (BSP) assay (T = tumor). The error bars represent the mean ± SD of 3 independent experiments, ** *P* < 0.01

Mutation, deletion, and epigenetic changes are the most common factors in the inactivation of tumor suppressor genes. The results of the bioinformatics analysis showed that the mutant rate of DMTN was 1.4% (1/72) in the colorectal (Genentech) databases, and the deletion rate of DMTN was 6.3% (37/589) and 2% (5/257) in the TCGA (Provisional) and TCGA (Nature 2012) CRC databases, respectively (Fig. 5a and Additional file 1: Figure S4A). These results suggested the participation of other factors in the downregulation of DMTN. Therefore, we further analyzed the methylation status of the DMTN promoter. The analysis results from the TGCA and GEO data showed that the degree of methylation of the DMTN gene promoter was significantly higher than that in the normal intestinal tissues (Additional file 1: Figure S4B and S4C). We used 5-Aza-CdR (demethylation drugs) to treat the CRC cells and further observed changes in DMTN expression. The western blot and RT-PCR results revealed an increase in the expression of DMTN in CRC cells treated with 5-Aza-CdR in a concentration-dependent manner (Fig. 5b, *P* < 0.05).

Then, we analyzed CpG islands in the DMTN gene using the UCSC database (http://genome.ucsc.edu/). The prediction results revealed two CpG islands, one in the promoter region and another in the first exon of the DMTN gene (Additional file 1: Figure S5A). The GC contents of the two CpG islands were 72 and 71.2%, the sizes were 250 bp and 793 bp, the ratios of the observed to the expected CpG were 0.89 and 0.62, and the CpG numbers were 20 and 89, respectively (Additional file 1: Figure S5B). Next, we detected the degree of methylation of the two CpG islands in the DMTN gene in 10 fresh CRC tissues using the bisulfite genomic sequence (BSP) assay. The results suggested a higher degree of CpG island methylation in the DMTN promoter in CRC tissues than in normal intestinal tissues (Fig. 5c, *P* < 0.05). Furthermore, combining the RT-PCR and bisulfite genomic sequence (BSP) assay results, we found a negative correlation between the expression of DMTN and the degree of methylation in the promoter (Fig. 5d, *P* < 0.05), and the expression of DMTN decreased with an increasing degree of promoter methylation (Fig. 5e). The TCGA data analysis showed the same result (Additional file 1: Figure S5C, *P* < 0.05).

## Discussion

It is necessary to identify genes with required expression for the proliferation or survival of tumor cells, and cancer dependencies represent targets for therapeutic efforts [13]. DMTN is a transcriptional differentially expressed gene (DEG) that was identified using CRC mRNA sequencing data, but little research has been conducted to examine the relationship between the abnormal expression of DMTN and tumorigenesis. Our results showed that DMTN was significantly downregulated in CRC tissues, and its expression level was closely related to advanced progression and a poor prognosis in CRC patients. The expression pattern suggested that DMTN might function as a tumor suppressor gene during the carcinogenesis and progression of CRC. Consistent with our results, Lutchman, M, et al. observed a frequent loss of DMTN in prostate cancer, even for metastatic high-grade prostate cancer, using fluorescent in situ hybridization (FISH), and concluded that the role of DMTN in tumorigenesis may be similar to that of the tumor suppressor gene NF2 in neurofibromatosis based on DMTN functional studies [32].

Metastasis is a very complex biological process that involves a decrease in adhesion between tumor cells, an increase in cell migration ability, and a reconfiguration of the cytoskeleton, among others. DMTN plays an important role in maintaining cell morphological integrity and regulating cell movement. Mohseni, M. et al. found that the migration speed of mouse embryonic fibroblasts (MEFs) with DMTN HP domain knockout (HPKO) was significantly slowed, and the skin wound healing of the HPKO mice was delayed [15]. However, fewer reports have addressed the effect of DMTN dysregulation on the invasion and metastasis of tumor cells. Our results showed that the overexpression of DMTN inhibited the migration and metastasis of CRC cells, while the knockdown of DMTN promoted tumor cell migration and metastasis.

Rho GTP family proteins belong to the Ras superfamily, and GTPase activity is regulated by GEF, GDI and GAP [19, 38, 39]. Rac1 is an important member of the Rho GTPase family, and the abnormal activation of Rac1 is closely related to initiation, invasion and metastasis in a large number of tumors [17, 40, 41]. Our previous GSEA assay results suggested that the “RHO_GTPASES” and “Rac1 signaling pathways” gene sets were significantly enriched in CRC tissues with low DMTN expression. Moreover, recent studies have also demonstrated that DMTN regulates the activity of Rho GTPase. Mohseni, M et al. found that the headpiece domain of DMTN regulates cell shape, motility, and wound healing by modulating RhoA activation [14, 15]. Lutchman, M, et al. revealed that DMTN interacts with the Ras-guanine nucleotide exchange factor Ras-GRF2 and modulates mitogen-activated protein kinase pathways, and the expression level of the active form of Rac1 (Rac1-GTP) was also slightly increased after the knockdown of DMTN [16]. However, the specific molecular mechanism of DMTN downregulation in the regulation of Rac1 signaling pathway activity remains unclear.

The tumor suppressor APC is mutated in sporadic and familial colorectal tumors, and wild-type APC interacts with the Rac1-specific guanine–nucleotide exchange factor (GEF) Asef and Asef2. In contrast, the truncated mutant APC activates Asef and Asef2 and then induces an increase in the levels of the active forms of Rac1 and Cdc42 to regulate cell adhesion and migration [33, 34, 42, 43]. Interestingly, our study showed that DMTN may play a similar role to APC during the invasion and metastasis of CRC. Our bioinformatics analysis and in vitro and in vivo experiments showed that DMTN inhibited the activity of Rac1 by interacting with ARHGEF2, but the downregulation of DMTN relieved the binding to the ARHGEF2 protein and then enhanced Rac1 signaling pathway activity, stimulated lamellipodia and protrusion formation, and promoted the CRC cell metastasis.

The inactivation of tumor suppressor genes is an important factor in tumorigenesis. The underlying mechanisms mainly consist of gene deletions and mutations, epigenetic changes, and posttranscriptional regulation. For example, numerous deletions and mutations of tumor suppressor genes, such as APC, p53, DCC, and PTEN, occur during the progression of CRC. Indeed, Lutchman, M, et al. have suggested a frequent loss of DMTN in prostate cancer [32]. However, our TGCA data analysis indicated that the deletion rate of DMTN was only approximately 6.3% in CRC tissues, which suggested the participation of other factors in the downregulation of DMTN. It is well known that the hypermethylation of tumor suppressor genes is an important factor leading to the decline in their expression. We observed a higher degree of CpG island methylation in DMTN in CRC tissues than in normal intestinal mucosal tissues, and the degree of methylation of the CpG islands was negatively correlated with the expression level of DMTN. These results suggested that hypermethylation of the gene promoter might not be a major factor in the inactivation of DMTN.

## Conclusion

**Fig. 6:**
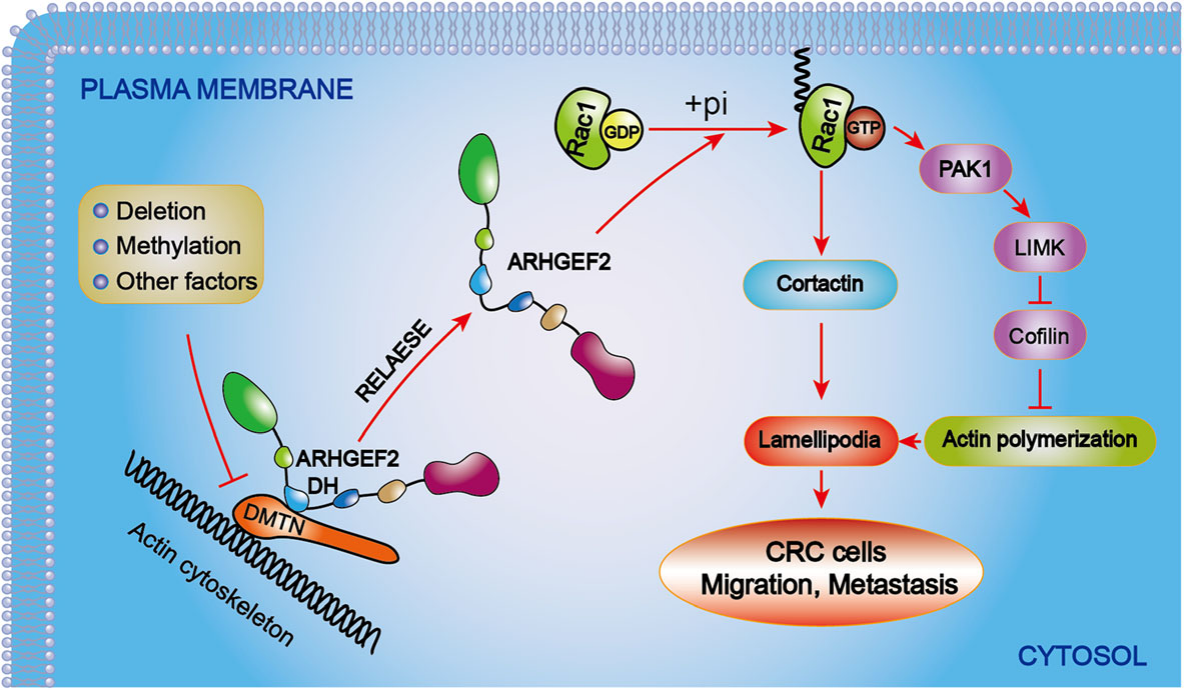
The mechanisms of DMTN in the invasion and metastasis of CRC. Hypermethylation and the deletion of DMTN relieved its binding to the ARHGEF2 protein, activated the Rac1 signaling pathway, regulated actin cytoskeletal rearrangements, and finally promoted the invasion and metastasis of CRC cells

In conclusion, we found that the expression of DMTN was significantly reduced in CRC tissues compared with that in paired adjacent normal tissues and that the downregulation of DMTN was associated with advanced progression and poor survival, and it was regarded as an independent predictive factor for the prognosis of patients with CRC. Furthermore, the overexpression of DMTN inhibited the invasion and metastasis of CRC cells, while the knockdown of DMTN promoted tumor invasion and metastasis. Moreover, hypermethylation and the deletion of DMTN relieved binding to the ARHGEF2 protein, activated the Rac1 signaling pathway, regulated actin cytoskeletal rearrangements, and finally promoted the invasion and metastasis of CRC cells (Fig. 6). In this study, we uncovered a novel functional role for DMTN in regulating the invasion and metastasis of CRC, which might provide a new therapeutic target enabling cancer precision medicine for CRC patients.

## Additional file

Additional file 1: **Table S1.** Primer Sequences Used for vector construction (5′ to 3′). **Table S2.** Primer Sequences Used for RT-PCR (5′ to 3′). **Table S3.** Primer Sequences Used for Bisulfite genomic sequence (BSP) assay (5′ to 3′). **Table S4.** The relationship between the expression of DMTN and clinicopathological parameters. **Table S5.** Spearman correlation analysis between the expression of DMTN and Clinicopathologic Features. **Figure S1.** Down-regulation of DMTN was correlated with advanced progression and poorer prognosis of CRC. **Figure S2.** Exogenous DMTN knockdown promotes the metastasis of CRC cells, up-regulation of DMTN inhibited metastasis of CRC cells. **Figure S3.** Down-regulation of DMTN enhances the activity of the RAC1 signaling pathway by relieving the binding with ARHGEF2 protein. **Figure S4.** Epigenetic regulation of DMTN gene through changes in the methylation status of the gene promoter. **Figure S5.** The analysis of CpG Island of DMTN gene, and the relationship between the expression of DMTN and the degree of CpG Island methylation. (ZIP 4993 kb)

## Abbreviations

ARHGEF2: Rho-Rac guanine nucleotide exchange factor 2
BSP: Bisulfite genomic sequence
CIN: chromosomal instability
Co-IP: Co-Immunoprecipitation
CRC: Colorectal cancer
GAPs: GTPase activating proteins
GDIs: Guanine dissociation inhibitors
GEFs: Guanine nucleotide exchange factors
GSEA: Gene Set Enrichment Analysis
IHC: Immunohistochemistry
MSI: Microsatellite instability
RT-PCR: Real-time quantitative PCR
TCGA: The Cancer Genome Atlas
WB: Western blotting
