# Supplementary material for: Hypermethylation of DMTN promotes the metastasis of colorectal cancer cells by regulating the actin cytoskeleton through Rac1 signaling activation
Source: J Exp Clin Cancer Res. 2018 Dec 4;37:299. doi: 10.1186/s13046-018-0958-1 (PMC6277997; doi:10.1186/s13046-018-0958-1)
Supplement: Supplementary file 1 — Table S1. Primer Sequences Used for vector construction (5′ to 3′). Table S2. Primer Sequences Used for RT-PCR (5′ to 3′). Table S3. Primer Sequences Used for Bisulfite genomic sequence (BSP) assay (5′ to 3′). Table S4. The relationship between the expression of DMTN and clinicopathological parameters. Table S5. Spearman correlation analysis between the expression of DMTN and Clinicopathologic Features. Figure S1. Down-regulation of DMTN was correlated with advanced progression and poorer prognosis of CRC. Figure S2. Exogenous DMTN knockdown promotes the metastasis of CRC cells, up-regulation of DMTN inhibited metastasis of CRC cells. Figure S3. Down-regulation of DMTN enhances the activity of the RAC1 signaling pathway by relieving the binding with ARHGEF2 protein. Figure S4. Epigenetic regulation of DMTN gene through changes in the methylation status of the gene promoter. Figure S5. The analysis of CpG Island of DMTN gene, and the relationship between the expression of DMTN and the degree of CpG Island methylation. (ZIP 4993 kb) [file 13046_2018_958_MOESM1_ESM.zip › Supplementary material_DMTN.docx]

**Supplementary Materials and Methods**

**Vectors construction and retroviral infection**

The DMTN over-expresion vector construction was generated by sub-cloning PCR-amplified full-length human DMTN cDNA into plasmid PSIN-EF2-puro. To knock out endogenous DMTN, 2 short hairpin RNA (shRNA) oligonucleotides (Supplementary Table S1) were respectively cloned into the pSuper-retro-puro vector to generate pSuper-retro-DMTN-RNAi(s). Retroviral production and infection were performed as previously described ^1^. Stable cell lines of DMTN overexpression or of DMTN knockdown were selected with 0.5 μg/ml puromycin in medium for 10 days.

**Real-time Quantitative PCR and western blotting**

Total RNA extraction, Real-time Quantitative PCR (RT-PCR) and western blotting were performed as previously described^1^. The primers used are shown in Supplementary Table S2. Anti-DMTN, anti-ARHGEF2 (Abcam, Cambridge, MA, USA), anti-Rac1, anti-PAK, anti-p-PAK, anti-LIMK, anti-p-LIMK, anti-Cotactin, anti-p-Cotactin, anti-Cofilin, anti-p-Cofilin (Cell Signaling Technology, Danvers, MA, USA) were used to detected corresponding proteins. A mouse anti-α-Tubulin monoclonal antibody (Sigma, Saint Louis, MO, USA) was used as a loading control.

**Immunohistochemistry**

The immunohistochemistry (IHC) were conducted according to previously described methods^1^. There are two index need to be observed. One is the proportion of positively stained tumor cells and the other is the strength of staining. These sections were scored respectively by two observers. The ratios of positive tumor cells were scored as follows: 0 (no positive tumor cells), 1 (<10% positive tumor cells), 2 (10%–50% positive tumor cells), and 3 (>50% positive tumor cells). The strength of staining was graded as following criteria: 0 (no staining), 1 (weak staining = light yellow), 2 (moderate staining = yellow brown), and 3 (strong staining = brown). The staining index (SI) was counted as staining strength score times ratio of positive tumor cells. Using this method of estimation, the expression of DMTN was scored as 0, 1, 2, 3, 4, 6 and 9. Cut-off values for DMTN were chosen in accordance with a measure of heterogeneity by the log-rank test statistical analysis in regard to overall survival. An optimal critical value was identified: ≤ 3 as low expression of DMTN, and the score of ≥ 4 was used to define tumors as high DMTN expression.

**Transwell Assay**

1×10^5^ Cells were plated on the top side of Matrigel-coated Boyden chamber (BD, Bedford, MA) and incubated at 37°C for 24-48 hours. Invaded and migratory cells on the lower membrane surface were fixed in 4% paraformaldehyde for 30 minutes. After removal of cells inside the upper chamber with cotton swabs, the invaded and migratory cells were stained with Giemsa for counting (3 random 100× fields per well). Cell counts were expressed as the mean number of cells per field of view. Three independent experiments were performed, and the data are presented as mean ± SD.

**Wound-healing assay**

CRC Cells were seeded in sixwell plates and incubated under permissive conditions until 90% confluence. After serum starvation for 24 h, wounds were created in the confluent cells using a pipette tip. Wound healing within the scrape line was then observed and photographed at indicated time points. Each experiment was repeated at least three times.

**Three-dimensional morphogenesis assay**

Twenty-four-well plates were coated with Growth Factor Reduced Matrige (BD Biosciences). Cells (1 × 10^4^ per well) suspended in growth medium containing 2% Matrigel were added on the top of the solidified Matrigel, and medium was replaced with 2% Matrigel every 3 to 4 days. Threedimensional morphological structure was observed and pictures were taken by a microscopy at 2-day intervals for 2–3 weeks. The filopodia formed by each cell sphere were counted according to the previous study.

**Orthotopic mouse metastatic model**

A surgical orthotopic implantation mouse model of CRC was performed as previously described^2^. Cells (2×10^6^ per mouse) were subcutaneously injected into the right dorsal flank of female BALB/c athymic nude mice (4–6 weeks of age, 18–20g) obtained from the Animal Center of Southern Medical University, Guangzhou, China. Two weeks later, animals were sacrificed and tumors were excised. A part of tumor was fixed in 10% formaldehyde, paraffin embedded, and then 5 µm sections were cut and subjected to hematoxylin-eosin (H&E). Another part of tumor was divided into small pieces approximately 1mm diameter. Surgical orthotopic implantation of the CRC tumor fragments was performed in nude mice after anesthesia. The mice were killed 100days after surgery, and the individual organs were excised and metastases were observed by histological analysis. The numbers of gross metastatic foci were determined using a dissection microscope. All of the mice used in this study were kept under specific pathogen-free conditions. All animal experiments were approved by the institutional Use Committee for Animal Care in accordance with standard procedures.

**References**

1 Liao WT, Jiang D, Yuan J, Cui YM, Shi XW, Chen CM *et al*. HOXB7 as a prognostic factor and mediator of colorectal cancer progression. Clin Cancer Res 2011; 17: 3569-3578.

2 Tseng W, Leong X, Engleman E. Orthotopic mouse model of colorectal cancer. Journal of visualized experiments : JoVE 2007: 484.

**Supplementary Table S1. Primer Sequences Used for vector construction (5' to 3')**

| Gene | Sense primer（5'→3'） | Anti-sense primer（5'→3'） |
| --- | --- | --- |
| DMTN over-expression | ACGCGGATCCGCCATGGATTACAAGGATGACGACGATAAGATGGAACGGCTGCAGAAGCAAC | GCCGGAATTCTCAGAAGAGAGAGGCCTTCTTC |
| DMTN shRNA1 | CGCGTCCGCGGAATGAGCTCAAGAAGAATTCAAGAGATTCTTCTTGAGCTCATTCCGCTTTTTGGAAAT | CGATTTCCAAAAAGCGGAATGAGCTCAAGAAGAATCTCTTGAATTCTTCTTGAGCTCATTCCGCGGA |
| DMTN shRNA1 | CGCGTCCGCCCATCTATAAGCAGAGAGATTCAAGAGATCTCTCTGCTTATAGATGGGCTTTTTGGAAAT | CGATTTCCAAAAAGCCCATCTATAAGCAGAGAGATCTCTTGAATCTCTCTGCTTATAGATGGGCGGA |

**Supplementary Table S2. Primer Sequences Used for RT-PCR (5' to 3')**

| Gene | Sense primer（5'→3'） | Anti-sense primer（5'→3'） |
| --- | --- | --- |
| DMTN | CGGTCGCCTGGAATCATCTC | CCACGGACTCTCTCTGCTT |
| GAPDH | ACAGTCAGCCGCATCTTCTT | GACAAGCTTCCCGTTCTCAG |

**Supplementary Table S3. Primer Sequences Used for Bisulfite genomic sequence (BSP) assay**

**(5' to 3')**

| Gene | Sense primer（5'→3'） | Anti-sense primer（5'→3'） |
| --- | --- | --- |
| DMTN CpG | CTCTTGTTTGAAATGGATTGAGC | CTTCCCCCAGGAGCCTAGGTAATA |

**Supplementary Table S4.** The relationship between the expression of DMTN and clinicopathological parameters

| Characteristics | DMTN expression | | Z | P value |
| --- | --- | --- | --- | --- |
|  | Low | High |  |  |
| Age |  |  |  |  |
| ≤mean (57) | 50 | 50 | 0.000 | 1.000 |
| >mean (57) | 50 | 50 |  |  |
| Gender |  |  |  |  |
| Male | 45 | 55 | -1.411 | 0.158 |
| Female | 55 | 45 |  |  |
| Differentiation |  |  |  |  |
| Well | 6 | 16 | -2.270 | 0.023 |
| moderate | 70 | 68 |  |  |
| poor | 24 | 16 |  |  |
| Dukes stage |  |  |  |  |
| Dukes A | 8 | 30 | -4.642 | 0.000 |
| Dukes B | 25 | 32 |  |  |
| Dukes C | 28 | 20 |  |  |
| Dukes D | 39 | 18 |  |  |
| T classification |  |  |  |  |
| T1 | 5 | 11 | -4.611 | 0.000 |
| T2 | 9 | 28 |  |  |
| T3 | 49 | 47 |  |  |
| T4 | 37 | 14 |  |  |
| N classification |  |  |  |  |
| N0 | 47 | 67 | -2.775 | 0.006 |
| N1 | 40 | 25 |  |  |
| N2 | 13 | 8 |  |  |
| M classification |  |  |  |  |
| M0 | 61 | 82 | -3.281 | 0.001 |
| M1 | 39 | 18 |  |  |

**Supplementary Table S5. Spearman correlation analysis between the expression of DMTN and Clinicopathologic Features**

| Variables | DMTN expression | |
| --- | --- | --- |
|  | Spearman correlation | P-value |
| Age | 0.000 | 1.000 |
| Gender | -0.100 | 0.159 |
| Differentiation | -0.161 | 0.023 |
| Dukes stage | -0.329 | 0.000 |
| T classification | -0.327 | 0.000 |
| N classification | -0.197 | 0.005 |
| M classification | -0.233 | 0.001 |
| Metastasis | -0.300 | 0.000 |

**Supplementary figure legends**

**Supplementary Figure S1. Down-regulation of DMTN was correlated with advanced progression and poorer prognosis of CRC.**

A, B, The analysis of the mRNA and protein expression of DMTN in CRC tissues and normal colon tissues using Oncomine database and The Human Protein Atlas; C, RT-PCR analysis of DMTN mRNA expression in 10 fresh human CRC tissues; D, Kaplan–Meier analyses of CRC patients outcome with low versus high expression of DMTN in GSE17536, GSE17537 and GSE16125 (P < 0.05, log-rank test).

**Supplementary Figure S2. Exogenous DMTN knockdown promotes the metastasis of CRC cells, up-regulation of DMTN inhibited metastasis of CRC cells.**

A, RT-PCR and Westernblot analysis of DMTN expression in CRC cell lines; B, C, D, Transwell invasion assay, Scratch wound healing and 3-D cell culture analysis of migration in CRC cells with DMTN overexpression or knockdown. Error bars represent the mean ± SD of 3 independent experiments, ** P < 0.01

**Supplementary Figure S3. Down-regulation of DMTN enhances the activity of the RAC1 signaling pathway by relieving the binding with ARHGEF2 protein.**

A, GSEA analysis of "RHO_GTPASES signaling pathways" gene set in low versus high expression group of DMTN in CRC; B, The analysis of the mRNA and protein expression of ARHGEF2 in CRC tissues and normal colon tissues using TCGA mRNA sequencing data.

**Supplementary Figure S4. Epigenetic regulation of DMTN gene through changes in the methylation status of the gene promoter.**

A, The analysis of deletion rate of DMTN in CRC using TCGA sequencing data; B, C, The analysis of the degree of DMTN promotor methylation in TGCA data and GEO data (GSE39334).

**Supplementary Figure S5.** **The analysis of CpG Island of DMTN gene, and the relationship between the expression of DMTN and the degree of CpG Island methylation.**

A, B, The analysis of CpG Island of DMTN gene; C, The relationship between the expression of DMTN and the degree of DMTN promotor methylation in TGCA data.
