# Supplementary figures and images for: Hypermethylation of DMTN promotes the metastasis of colorectal cancer cells by regulating the actin cytoskeleton through Rac1 signaling activation
Source: J Exp Clin Cancer Res. 2018 Dec 4;37:299. doi: 10.1186/s13046-018-0958-1 (PMC6277997; doi:10.1186/s13046-018-0958-1)

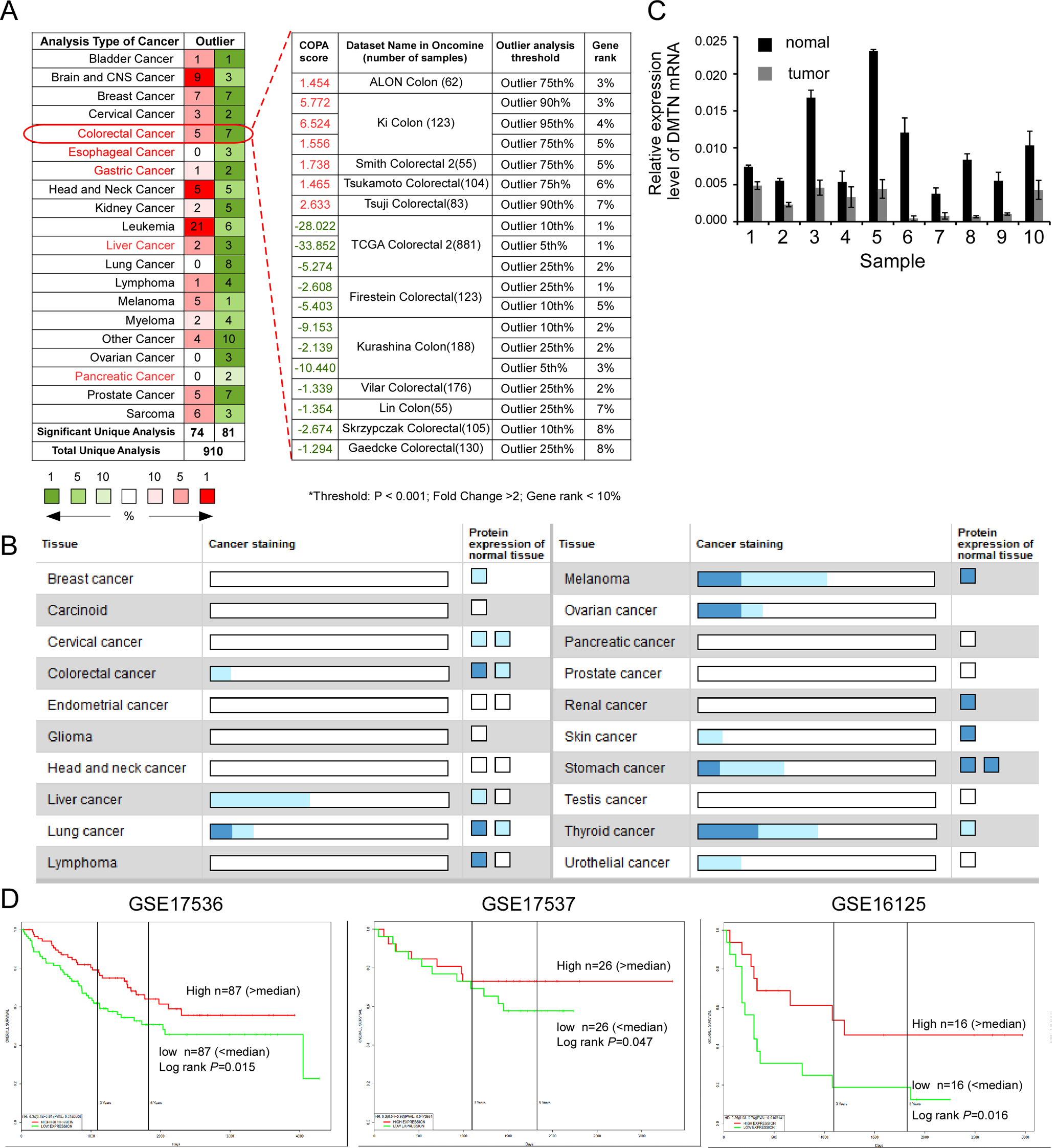

Supplement: Supplementary file 1 — Table S1. Primer Sequences Used for vector construction (5′ to 3′). Table S2. Primer Sequences Used for RT-PCR (5′ to 3′). Table S3. Primer Sequences Used for Bisulfite genomic sequence (BSP) assay (5′ to 3′). Table S4. The relationship between the expression of DMTN and clinicopathological parameters. Table S5. Spearman correlation analysis between the expression of DMTN and Clinicopathologic Features. Figure S1. Down-regulation of DMTN was correlated with advanced progression and poorer prognosis of CRC. Figure S2. Exogenous DMTN knockdown promotes the metastasis of CRC cells, up-regulation of DMTN inhibited metastasis of CRC cells. Figure S3. Down-regulation of DMTN enhances the activity of the RAC1 signaling pathway by relieving the binding with ARHGEF2 protein. Figure S4. Epigenetic regulation of DMTN gene through changes in the methylation status of the gene promoter. Figure S5. The analysis of CpG Island of DMTN gene, and the relationship between the expression of DMTN and the degree of CpG Island methylation. (ZIP 4993 kb) [file 13046_2018_958_MOESM1_ESM.zip › Figure S1.tif]

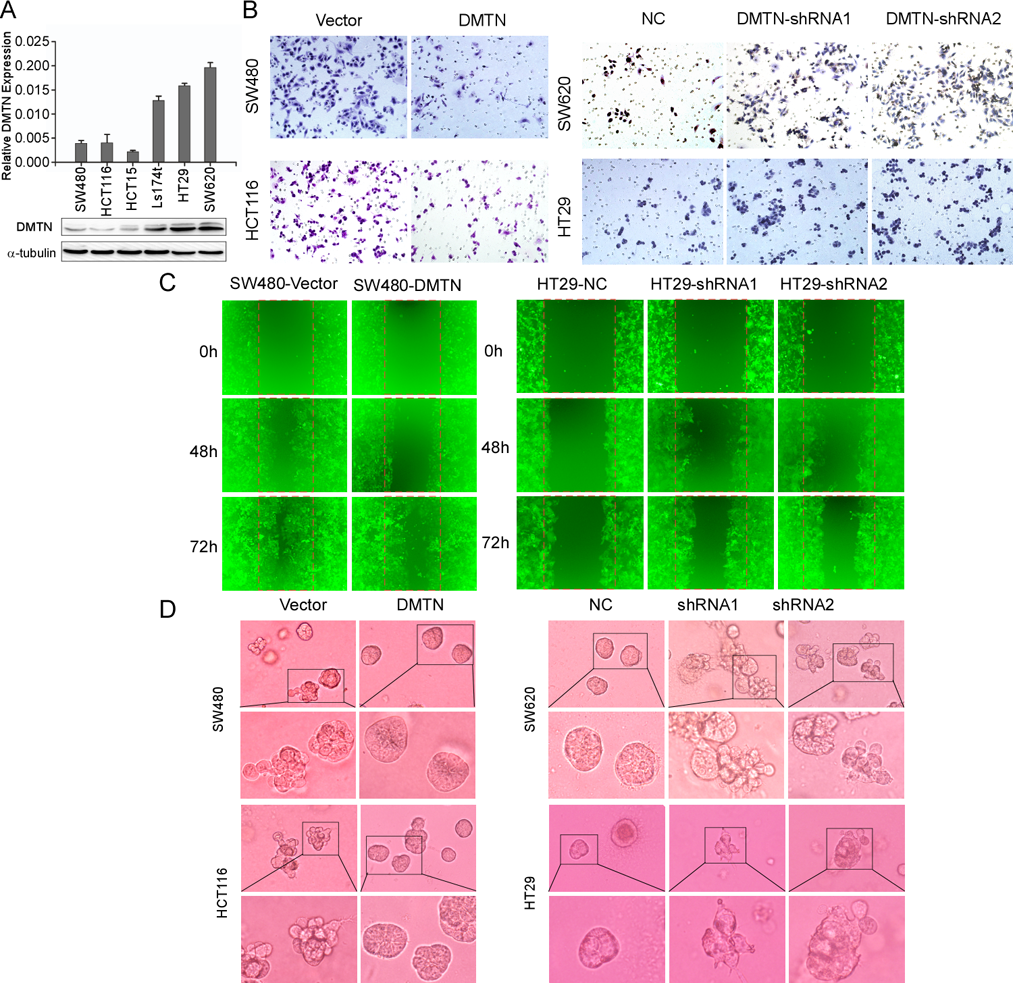

Supplement: Supplementary file 1 — Table S1. Primer Sequences Used for vector construction (5′ to 3′). Table S2. Primer Sequences Used for RT-PCR (5′ to 3′). Table S3. Primer Sequences Used for Bisulfite genomic sequence (BSP) assay (5′ to 3′). Table S4. The relationship between the expression of DMTN and clinicopathological parameters. Table S5. Spearman correlation analysis between the expression of DMTN and Clinicopathologic Features. Figure S1. Down-regulation of DMTN was correlated with advanced progression and poorer prognosis of CRC. Figure S2. Exogenous DMTN knockdown promotes the metastasis of CRC cells, up-regulation of DMTN inhibited metastasis of CRC cells. Figure S3. Down-regulation of DMTN enhances the activity of the RAC1 signaling pathway by relieving the binding with ARHGEF2 protein. Figure S4. Epigenetic regulation of DMTN gene through changes in the methylation status of the gene promoter. Figure S5. The analysis of CpG Island of DMTN gene, and the relationship between the expression of DMTN and the degree of CpG Island methylation. (ZIP 4993 kb) [file 13046_2018_958_MOESM1_ESM.zip › Figure S2.tif]

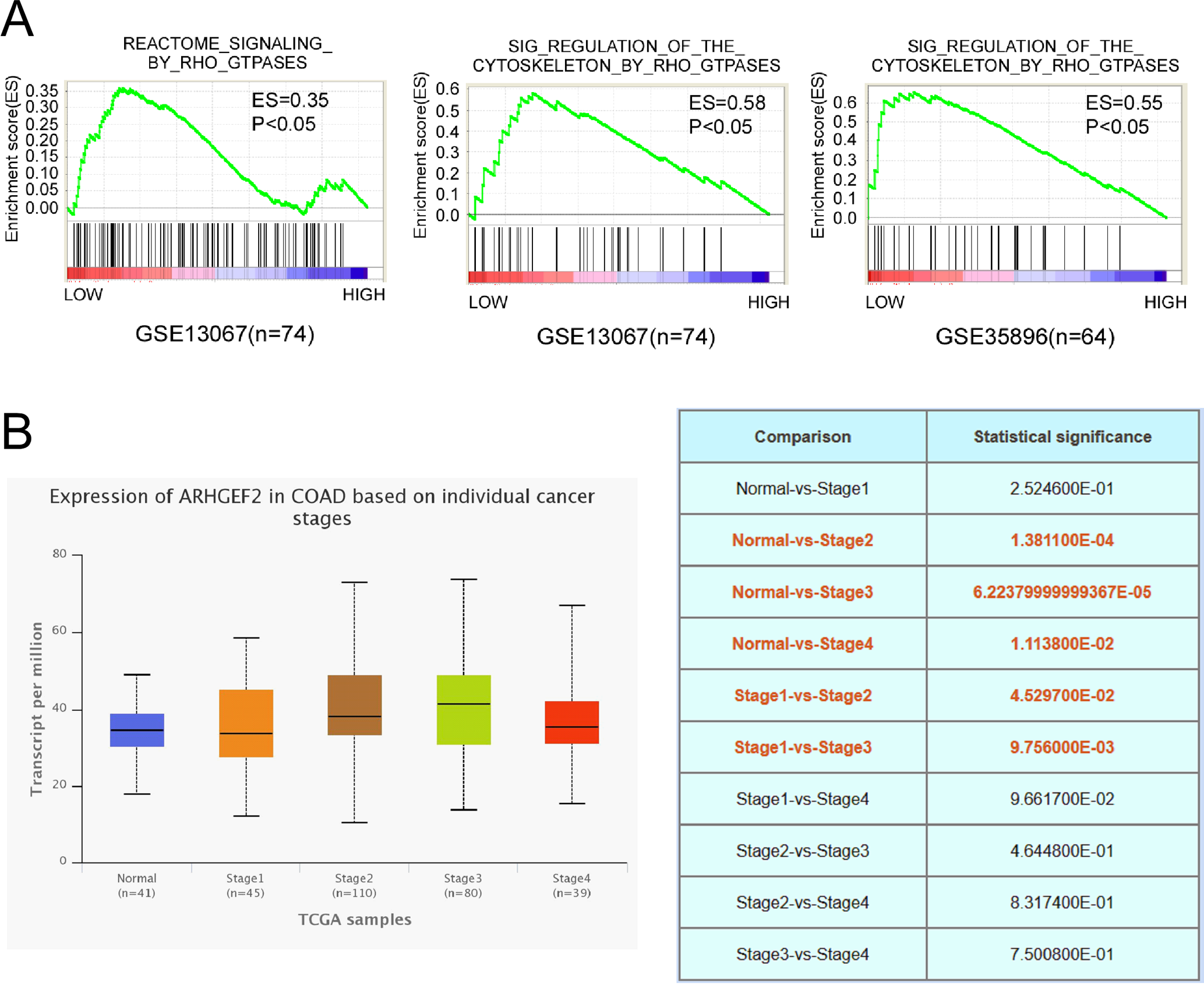

Supplement: Supplementary file 1 — Table S1. Primer Sequences Used for vector construction (5′ to 3′). Table S2. Primer Sequences Used for RT-PCR (5′ to 3′). Table S3. Primer Sequences Used for Bisulfite genomic sequence (BSP) assay (5′ to 3′). Table S4. The relationship between the expression of DMTN and clinicopathological parameters. Table S5. Spearman correlation analysis between the expression of DMTN and Clinicopathologic Features. Figure S1. Down-regulation of DMTN was correlated with advanced progression and poorer prognosis of CRC. Figure S2. Exogenous DMTN knockdown promotes the metastasis of CRC cells, up-regulation of DMTN inhibited metastasis of CRC cells. Figure S3. Down-regulation of DMTN enhances the activity of the RAC1 signaling pathway by relieving the binding with ARHGEF2 protein. Figure S4. Epigenetic regulation of DMTN gene through changes in the methylation status of the gene promoter. Figure S5. The analysis of CpG Island of DMTN gene, and the relationship between the expression of DMTN and the degree of CpG Island methylation. (ZIP 4993 kb) [file 13046_2018_958_MOESM1_ESM.zip › Figure S3.tif]

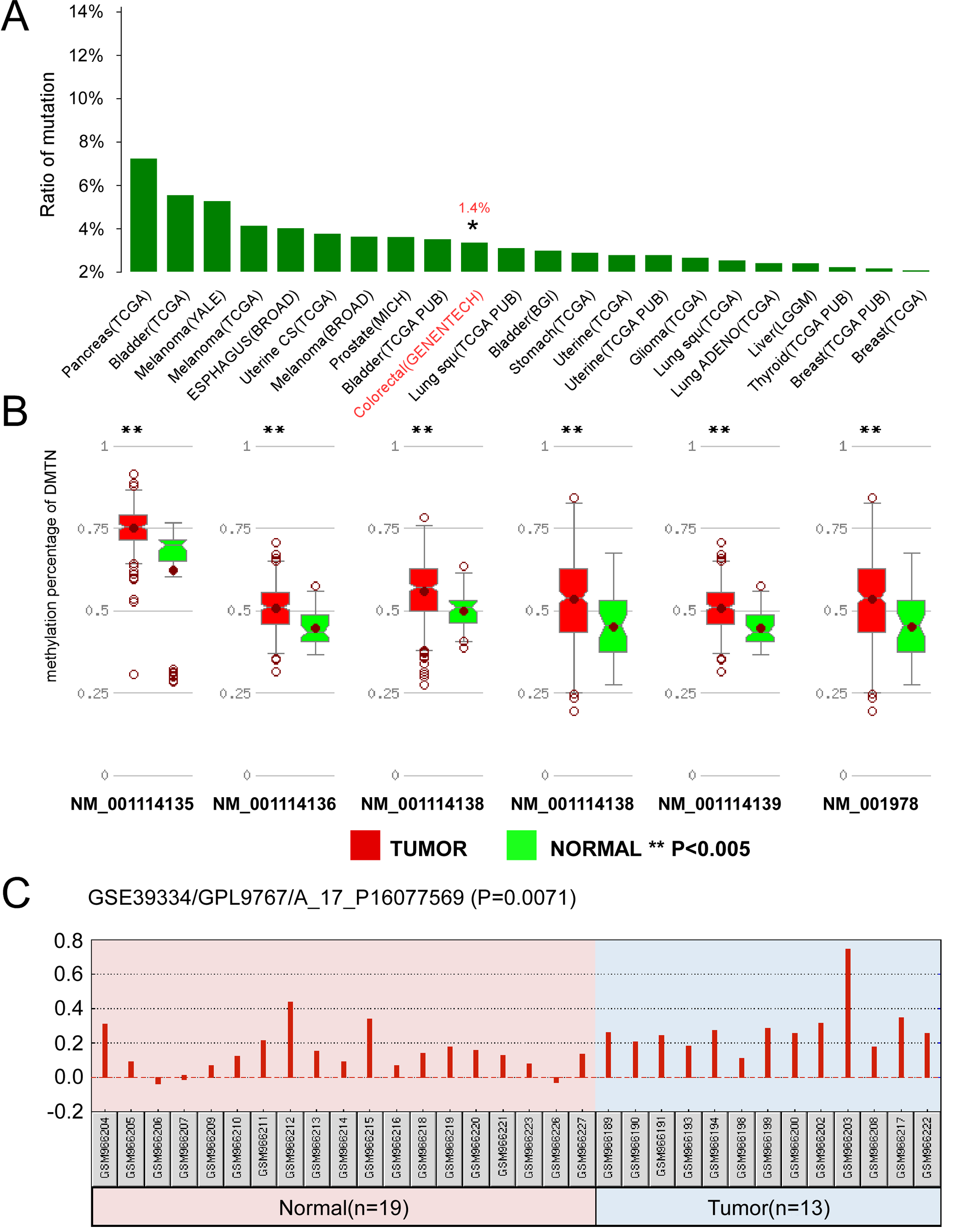

Supplement: Supplementary file 1 — Table S1. Primer Sequences Used for vector construction (5′ to 3′). Table S2. Primer Sequences Used for RT-PCR (5′ to 3′). Table S3. Primer Sequences Used for Bisulfite genomic sequence (BSP) assay (5′ to 3′). Table S4. The relationship between the expression of DMTN and clinicopathological parameters. Table S5. Spearman correlation analysis between the expression of DMTN and Clinicopathologic Features. Figure S1. Down-regulation of DMTN was correlated with advanced progression and poorer prognosis of CRC. Figure S2. Exogenous DMTN knockdown promotes the metastasis of CRC cells, up-regulation of DMTN inhibited metastasis of CRC cells. Figure S3. Down-regulation of DMTN enhances the activity of the RAC1 signaling pathway by relieving the binding with ARHGEF2 protein. Figure S4. Epigenetic regulation of DMTN gene through changes in the methylation status of the gene promoter. Figure S5. The analysis of CpG Island of DMTN gene, and the relationship between the expression of DMTN and the degree of CpG Island methylation. (ZIP 4993 kb) [file 13046_2018_958_MOESM1_ESM.zip › Figure S4.tif]

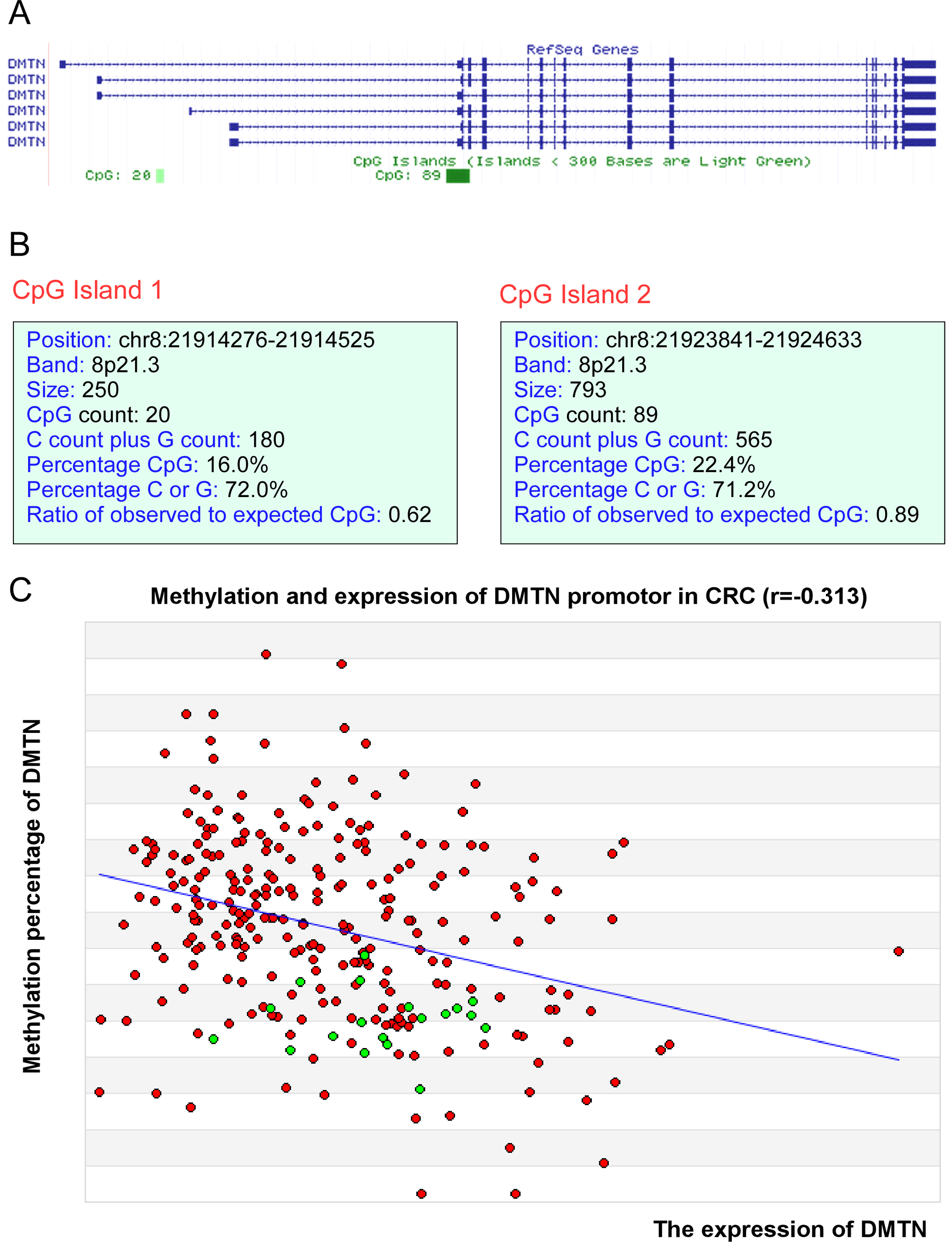

Supplement: Supplementary file 1 — Table S1. Primer Sequences Used for vector construction (5′ to 3′). Table S2. Primer Sequences Used for RT-PCR (5′ to 3′). Table S3. Primer Sequences Used for Bisulfite genomic sequence (BSP) assay (5′ to 3′). Table S4. The relationship between the expression of DMTN and clinicopathological parameters. Table S5. Spearman correlation analysis between the expression of DMTN and Clinicopathologic Features. Figure S1. Down-regulation of DMTN was correlated with advanced progression and poorer prognosis of CRC. Figure S2. Exogenous DMTN knockdown promotes the metastasis of CRC cells, up-regulation of DMTN inhibited metastasis of CRC cells. Figure S3. Down-regulation of DMTN enhances the activity of the RAC1 signaling pathway by relieving the binding with ARHGEF2 protein. Figure S4. Epigenetic regulation of DMTN gene through changes in the methylation status of the gene promoter. Figure S5. The analysis of CpG Island of DMTN gene, and the relationship between the expression of DMTN and the degree of CpG Island methylation. (ZIP 4993 kb) [file 13046_2018_958_MOESM1_ESM.zip › Figure S5.tif]
